# Music use under academic stress: university students’ experiences of emotion regulation and learning re-engagement

**DOI:** 10.3389/fpsyg.2026.1989359

**Published:** 2026-10-26

**Authors:** Leiyu Tao, Xueping Xing

**Affiliations:** 1 School of Design, Jiangsu Open University, Nanjing, China; 2 Office of Research Administration, Weifang Nursing Vocational College, Qingzhou, China

**Keywords:** academic stress, emotion regulation, learning re-engagement, music listening, university students

## Abstract

Academic stress can disrupt emotion, attention and persistence, yet little is known about how students use music at the moment between becoming stuck and returning to a task. This study explored when and why university students intentionally used self-selected music, how they understood its helpful or disruptive processes, and which conditions shaped perceived learning re-engagement. Fifteen undergraduates from two universities in eastern China completed Mandarin semi-structured critical-incident interviews about helpful and counterproductive episodes. Reflexive thematic analysis, sensitised by the extended process model of emotion regulation and task-level self-regulated learning, generated four themes. Students gave music jobs at moments of friction; described bodily or attentional shifts that preceded renewed academic action; judged usefulness through the fit among music, task, person and setting; and developed rules for monitoring, stopping or choosing silence. Music was experienced as a conditional bridge towards action, but could also accompany distraction, avoidance or overload. These retrospective accounts generate propositions about goals, task fit and self-monitoring among existing music users; they establish neither objective learning benefits nor the effectiveness of music-use guidance.

## 1 Introduction

A deadline is approaching. A university student opens an assignment, reads the same sentence three times, and feels attention slide towards worry. Instead of contacting a counsellor or abandoning the task, the student reaches for headphones. This ordinary scene matters because academic stress is not confined to examinations: workload, uncertainty, difficult feedback, competing roles, and fear of underperformance can unsettle emotion, attention, and persistence across a semester. Higher academic stress is associated with poorer mental well-being among university students, while review evidence links stress with impaired learning and educational functioning (Barbayannis et al., 2022; Pascoe et al., 2020). Yet the small actions students take between feeling overwhelmed and attempting the next paragraph remain comparatively underexamined.

Music is one such everyday action. Here, intentional use of self-selected music means deliberately choosing, retaining or adjusting music to address a perceived difficulty or desired state around an academic task. It includes selecting a familiar playlist without choosing every track and purposefully keeping music in the background; it excludes incidental exposure without a regulatory purpose. A deliberate response did not require a fully articulated goal at the time. Background listening describes music’s position relative to the task, whereas intentionality concerns the listener’s purpose. During the COVID-19 disruption, students reported music listening as a coping resource (Vidas et al., 2021, 2022). A broader scoping review identifies arousal regulation, mood management, cognitive processes, social connection and identity as plausible pathways linking musical activity with well-being (Dingle et al., 2021). These findings do not establish that pressing play reliably helps a student return to learning.

The evidence is mixed. Reviews of music interventions report benefits for stress-related outcomes and emotion regulation, whereas a meta-analysis focused on post-stressor listening in healthy participants found no significant overall effect on recovery (Adiasto et al., 2022; de Witte et al., 2020; Peters et al., 2024). Background-music research likewise reports beneficial, null and detrimental learning effects, with task demands and musical features among the likely moderators (de la Mora Velasco and Hirumi, 2020). Preferred music may increase reported task focus without improving performance (Kiss and Linnell, 2021). Treating music as uniformly therapeutic therefore conceals students’ decisions and the occasions on which listening fails.

This study connects those decisions to learning through a nested, episode-based framework. The extended process model treats emotion regulation as identifying a need to regulate, selecting and implementing a strategy, and monitoring its consequences (Gross, 2015; McRae and Gross, 2020). Music is considered a flexible tactic within that sequence. Three candidate triggers help specify its possible functions: bodily tension may make arousal regulation salient; repetitive checking may prompt interruption of attentional fixation; and environmental noise may favour acoustic shielding (Dingle et al., 2021; Harley et al., 2019). These triggers are prioritised analytically because they locate different immediate barriers to task action, not because their frequency or causal importance is known. Salient sound may instead add competing material and hinder the next task (de la Mora Velasco and Hirumi, 2020). A task-level self-regulated learning lens then asks whether students report renewed goal-directed attention, effort and persistence (Theobald, 2021; Zheng et al., 2023). Learning re-engagement therefore means perceived resumption or continuation of a specific academic task during or after intentional music use, rather than measured attainment.

Existing research rarely follows this complete pathway in students’ own accounts. Surveys relate listening tendencies to well-being or elicit music choices, whereas background-music studies commonly examine listening alongside a task (Adiasto et al., 2023; Goltz and Sadakata, 2021; Morgan and Marroquín, 2024). A qualitative study across Chinese universities examined intertwined learning and well-being uses without tracing a defined stress episode and subsequent task return (Hu et al., 2021). A meta-ethnography identified only seven qualitative studies of university students’ music listening for emotion regulation and highlighted intention, context and consequences (Lee et al., 2026). The overall objective of this study is to develop an episode-based account of how existing music users recognise academic friction, select and monitor music, and interpret its relationship with perceived learning re-engagement or continued disengagement. This study therefore asks: (RQ1) In which academic stress situations, and with what regulatory goals, do university students intentionally use music? (RQ2) How do students describe the emotional, attentional, and cognitive processes through which music supports—or hinders—the resumption and sustained engagement of academic tasks? (RQ3) What personal, musical, task-related, and situational conditions shape whether music use is followed by learning re-engagement or continued disengagement?

## 2 Literature review

### 2.1 Academic stress, emotion, and returning to the task

Academic stress arises when educational demands are appraised as taxing or exceeding available resources. Examinations, deadlines, feedback and accumulated workload can be associated with distress and poorer perceived well-being (Barbayannis et al., 2022; Pascoe et al., 2020). The relationship with learning is conditional: students interpret demands and regulate their responses, so similar events can produce different emotions and actions.

Emotion regulation can alter attention, task interpretation and persistence in achievement situations (Harley et al., 2019). Evidence nevertheless remains fragmented on how momentary emotion relates to ongoing self-regulation (Zheng et al., 2023). Although self-regulated learning training can improve strategy use, motivation and performance, this literature foregrounds planned strategies rather than informal tactics recruited when work stalls (Theobald, 2021). Examining the next reported academic action connects such tactics to task engagement without equating emotional relief with measured productivity or grades.

### 2.2 Music as self-directed emotion regulation

People use music to alter, maintain, understand or express affective states through processes including arousal adjustment, distraction, autobiographical memory and social connection (Dingle et al., 2021). A scoping review found that listening dominated music-based emotion-regulation studies, but definitions were inconsistent and music was often treated as a single independent variable (Chong et al., 2024). Selecting instrumental music to reduce agitation may serve a different goal from choosing an energising song or a familiar sad piece.

The extended process model distinguishes identifying a regulatory need, selecting and implementing a strategy, and monitoring its suitability (Gross, 2015; McRae and Gross, 2020). Music can participate in several processes: headphones alter the soundscape, a playlist redirects attention, and memories or musical intensity may change felt emotion. These are candidate functions, not fixed properties of genres. Longitudinal evidence links music-based regulation with subjective stress and coping, while qualitative synthesis describes sensory and reflective uses (Lee et al., 2026; Leipold et al., 2025). Neither intention nor relief, however, demonstrates a return to academic work.

Intervention reviews report benefits for some stress-related and emotion-regulation outcomes, but combine prescribed listening, active music-making and other activities that differ from everyday self-selection (de Witte et al., 2020; Peters et al., 2024). A meta-analysis of experimental listening studies found heterogeneous stress-recovery effects (Adiasto et al., 2022). An online survey eliciting music choices after a hypothetical stressor instead examined selection preferences, not observed recovery or learning (Adiasto et al., 2023). Observational studies further associate some music-regulation patterns with poorer well-being or emotional symptoms (Morgan and Marroquín, 2024; Tan et al., 2024; Vidas et al., 2022). Together, these findings warrant attention to rumination, distraction and avoidance alongside perceived benefit.

Algorithmically curated listening adds questions of trust, emotional autonomy and privacy to self-selection, but recent qualitative work on these issues did not trace the next action in a defined academic task (Zheng et al., 2026).

### 2.3 Music, attention, and studying: a conditional resource

Students report selecting music for concentration, mood and endurance (Goltz and Sadakata, 2021). Preferred background music increased reported task focus in a sustained-attention study, although this did not establish improved task performance (Kiss and Linnell, 2021). Such findings suggest a possible distinction between feeling focused and demonstrating learning gains.

Background sound also competes with the task. A review of 30 learning studies identified positive, neutral and negative findings, with incomplete reporting of musical characteristics limiting comparison (de la Mora Velasco and Hirumi, 2020). Under a stressful decision task, music increased cognitive effort and silence favoured performance (Arboleda et al., 2022). These results direct attention to task demands and musical salience. They say less about how students switch tracks, lower the volume or choose silence. Pre-task listening and concurrent listening therefore need to be distinguished: a selection experienced as emotionally helpful may interfere once the academic task begins.

### 2.4 An episode-based account of learning re-engagement

An episode-based account connects four analytically distinct processes. Emotion regulation targets an affective state; attentional regulation redirects or sustains focus; environmental management changes external conditions; and study-strategy adaptation changes how the academic problem is approached. Their co-occurrence does not make them interchangeable. They are examined together because students may coordinate them around the same immediate goal of returning to a task. Achievement-focused emotion regulation links affective goals with learning situations, while self-regulated learning directs attention to monitoring and subsequent action (Harley et al., 2019; Zheng et al., 2023). Emotional change is thus one possible route, not a required mediator of re-engagement.

Qualitative investigation can clarify what broad associations and average effects leave unresolved: how a particular difficulty becomes a listening decision, how that decision changes during an episode, and whether students distinguish relief from returning to work (Adiasto et al., 2022; Hu et al., 2021; Lee et al., 2026). Critical-incident interviews about helpful and counterproductive episodes address these questions while treating students as active but fallible interpreters of their own regulation.

## 3 Methodology

### 3.1 Research design and conceptual orientation

This study used an interpretivist, contextualist qualitative design to examine students’ accounts of intentional music use during academic stress and subsequent task engagement. Accounts were treated as situated meanings co-produced in interviews, not transparent records of one reality. This position was congruent with reflexive thematic analysis (RTA), in which researchers actively develop interpretations and remain accountable for their choices (Braun and Clarke, 2021a, 2022; Maher, 2025). The study explored perceived processes, not causal effects on stress or performance. The extended process model sensitised the analysis to identifying a regulatory need, selecting and implementing music, and monitoring its consequences (Gross, 2015; McRae and Gross, 2020). A task-level self-regulated learning lens directed attention to whether students resumed or continued a specific task, reinvested effort, persisted or changed strategy. These nested lenses informed interpretation without becoming fixed coding templates.

### 3.2 Setting, participants and sampling

Participants were undergraduates at two public universities in eastern China, anonymised as Universities A and B. Eligibility required being at least 18 years old, experiencing academic stress during 2025–2026, and intentionally using self-selected music in relation to that experience. Students were excluded if severe hearing impairment precluded self-selected listening or if they were receiving clinical music therapy or treatment for a mental disorder. Thus, the sample represented experienced music users, not undergraduates generally.

Purposive maximum-variation sampling sought diversity in gender, year, discipline, music training and listening habits (Ahmad and Wilkins, 2025). Student counsellors assisted recruitment between December 2025 and January 2026; no course assessor took part. Recruitment initially sought 20 participants and yielded 17 eligible volunteers. Two completed pilot interviews that were excluded after protocol refinement, leaving an analytic sample of 15 students (six men and nine women), aged 19–23 years (M = 20.60, SD = 1.24), across Years 1–4 and six disciplines. Table 1 retains only the characteristics needed to show maximum variation and interpret RQ3; combinations with greater disclosure risk are reported in aggregate.

**Table 1:** Selected, de-identified participant characteristics (*N* = 15).

Code	Year	Broad field	Music experience	Habitual timing around study
P01	1	STEM	None	Occasional, before
P02	1	STEM	None	Regular, during
P03	1	Humanities	None	Routine, before
P04	2	Social sciences/business	None	Regular, during
P05	2	STEM	None	Routine, before
P06	2	Social sciences/business	Formal training	Occasional, before
P07	2	STEM	None	Regular, during
P08	3	STEM	None	Before and during
P09	3	Social sciences/business	Formal training	Regular, during
P10	3	Humanities	None	Routine, before
P11	4	Arts	Specialist background	Before and during
P12	3	STEM	None	Occasional, during
P13	4	Social sciences/business	None	Routine, before
P14	4	Social sciences/business	None	Occasional, before
P15	2	Arts	Formal training	Regular, during

Age, gender and institutional site are reported in aggregate in the text. The six disciplines represented in the sample are grouped into four broad disciplinary fields to reduce deductive disclosure; music-experience categories were likewise broadened. Timing labels summarise screening responses and do not imply measured listening frequency.

Information power was used to appraise the adequacy of the resulting dataset by considering the focused aim, sample specificity, theoretical guidance, interview quality and account richness (Malterud et al., 2016). It was not represented as an iterative stopping rule. Consistent with RTA, absence of new codes was not used to claim saturation (Braun and Clarke, 2021b).

### 3.3 Data collection

The semi-structured protocol drew on the research questions, conceptual framework and music-regulation literature, was reviewed by two researchers with qualitative and music-psychology expertise, and was piloted with two eligible students (Adeoye-Olatunde and Olenik, 2021; Kallio et al., 2016). Piloting clarified wording, shortened event recall and added a prompt about unhelpful use. The revised critical-incident protocol (see Appendix A, Semi-structured Interview Protocol) reconstructed a recent episode before, during and after music use, then explored a mixed or counterproductive episode. It covered regulatory goals and selection (RQ1), perceived emotional, attentional and cognitive processes (RQ2), and personal, musical, task-related and situational conditions (RQ3).

The first author conducted Mandarin voice interviews via Tencent Meeting during March–April 2026. The guide anticipated 45–60 min; interviews lasted 42–63 min (mean = 51 min). The interviewer had systematic qualitative-methods and interviewing-skills training and no teaching, supervisory or prior relationship with participants. Participants gave audio-recorded verbal consent and agreed to recording. Field notes captured context and reflexive impressions. Closing summaries invited correction or elaboration but were not treated as validation of one ‘true’ account. No payment or course credit was provided, and no song files or lyrics were collected. A professional service produced verbatim transcripts and therefore accessed audio for transcription. Researchers checked every transcript against its recording and removed direct identifiers; identifiable materials were otherwise restricted to the core team.

### 3.4 Reflexive thematic analysis

The 15 complete transcripts were analysed during May–June 2026 through RTA’s six recursive phases: familiarisation, coding, initial theme construction, review, definition and naming, and writing (Braun and Clarke, 2021a, 2022). Pilot data were not coded. Both authors repeatedly read and coded the complete dataset, using NVivo 14 to organise—not generate—codes, extracts and memos. Initial coding was primarily inductive, experiential and semantic. Narrated academic-stress episodes were the principal unit of analytic attention, while case summaries preserved each participant’s wider account and shared meanings were developed across the full dataset.

Later interpretation was informed, but not predetermined, by the extended process model and task-level re-engagement lens. Analysts traced regulatory need, music selection and implementation, monitoring cues and subsequent academic action; one extract could receive several process codes. Helpful, mixed and counterproductive episodes were compared, and a participant-by-theme matrix supported examination of person–music–task–situation variation. Candidate themes were organised around central concepts and reworked against extracts, full transcripts and disconfirming cases. Memos and thematic maps documented decisions. Across eight meetings, the second author acted as a critical friend by challenging assumptions and theme boundaries; this sought reflexive depth, not inter-coder agreement. Appendix B presents the final coding framework and analytic trail as an outcome of RTA, not an *a priori* reliability tool.

### 3.5 Rigour, reflexivity and translation

Rigour rested on methodological coherence, reflexive openness and transparent documentation rather than coding-reliability tests (Braun and Clarke, 2021a; Maher, 2025). The first author was a faculty member in the School of Design and a frequent self-directed music listener; she had no teaching or supervisory relationship with the participants at either university. A reflexive journal examined how this familiarity could favour benefit-oriented readings. The audit trail retained protocol revisions, sampling decisions, pilot feedback, field notes, coding iterations, theme maps, meeting records and alternative interpretations. Critical-friend dialogue and attention to mixed and counterproductive cases interrogated the analysis and made the researchers’ interpretive positions visible (Olmos-Vega et al., 2023). RTARG guided RTA-specific reporting completeness rather than serving as a quality certificate (Braun and Clarke, 2024).

Interviews, transcription, coding and theme development occurred in Mandarin; English wording therefore did not determine the initial codes or themes. After themes stabilised, a translator with psychology training, TEM-8 certification and academic translation experience translated labels and selected quotations. A senior English-language editor experienced in music psychology checked these against Mandarin sources and audio. Differences were discussed for conceptual and affective equivalence (Yunus et al., 2022). Verification attended to the regulatory intention, sequence of events, emotional intensity and participant metaphors. For example, “open a channel” was retained rather than converted into a measured cognitive mechanism, and “work mode” conveyed the contextual meaning of the Mandarin expression for being in a particular state. Appendix C links the Mandarin excerpts, English renderings and translation decisions. This was source-based bilingual checking, not independent back-translation; anonymisation used shorter continuous excerpts and generalised contextual descriptions.

### 3.6 Ethical considerations

Before recruitment, the Ethics Review Working Group of the School of Design, Jiangsu Open University approved the study. Its filing procedure for non-interventional, minimal-risk humanities and social-science research assigned no study-specific number. Student counsellors assisted recruitment, while an interviewer with no teaching, supervisory or prior relationship with participants obtained recorded consent and conducted the interviews. No course assessor participated, and no payment or course credit was offered. Participation was voluntary: students could decline questions, pause or withdraw without penalty or explanation, and request data withdrawal within one week. These arrangements separated the research encounter from educational assessment and routine student support. The distress protocol centred on pausing, checking the participant’s preference about continuing, ending the interview on request and providing referral information (Whitney and Evered, 2022). The interviewer clarified that this was research, not counselling or music therapy. Reports omitted names, used P01–P15 and generalised potentially identifying details. The professional transcription service accessed recordings for transcription; beyond that access, identifiable material was restricted to the core research team, excluding recruitment counsellors. Audio and transcripts were stored separately on encrypted drives and scheduled for secure deletion after one year (Taquette and Borges da Matta Souza, 2022).

## 4 Findings

The analysis generated four themes: assigning music a specific job at a moment of academic friction; moving from bodily or attentional change to renewed task action; the conditional fit between music, task, person and setting; and learning to monitor, stop or choose silence. These themes represent how participants interpreted particular helpful and unhelpful episodes, rather than evidence that music caused the changes they recalled. Quotations were translated and checked through the bilingual process described in Section 3.5, with participant codes P01–P15 used throughout. Disconfirming cases defined the boundaries of all four themes, as specified below and documented in Appendix B.

### 4.1 Giving music a job at moments of friction

The accounts began at identifiable points of friction rather than general busyness: a simulation that would not run, a derivation that went in circles, an anomalous value, an empty document, or sources that would not form an argument. Irritation, heaviness or self-doubt accompanied repeated, unproductive actions. P14 kept inspecting the same regression output, while P13 repeatedly rearranged possible literature-review structures. Music became an option when continuing in the same manner no longer felt productive. The purpose was not always explicit at the time: P01 described the intended change more clearly in retrospect than during the episode. Theme 1 therefore concerns a perceived response to friction, without assuming fully articulated planning in every case.

The assigned job differed. P01, P05, P07, P08, P12 and P14 sought a brief interruption before looking again. P05 said that music did not provide the engineering insight; “it let me step back from that dead loop” (P05). P13 wanted to clear a mental desktop crowded by possibilities. P02 instead increased a familiar instrumental playlist to cover roommates’ voices, while P04 and P07 used headphones as an acoustic boundary. Such soundscape management was not always understood as emotion regulation:

*For me, it was less a form of “emotion regulation” and more a form of “environmental management.” I did not listen in order to become happy; I listened in order to* “block things out”*. It was the door through which I pulled myself out of the noisy physical world and into a space containing only the problem and me. Yet I also knew that the door could block the wrong thing, such as my own voice when I needed to recite. (P02)*

P03, P06, P10, P11 and P15 sought affective or perceptual attunement before writing or creative work. P10 chose a piano piece whose restrained mood resembled the novel under study; P06 used a familiar vocal performance to move from “technical mode” towards narrative explanation (P06). P05 wanted activation from familiar rock rather than soothing. For P11, attentive listening to another work was a way of reopening possibilities. These contrasts, especially P02’s environmental goal and P05’s activation goal, bounded Theme 1 around assigning a particular job rather than assuming that all music use sought emotional calming.

### 4.2 Music was experienced as a transition; students did the academic work

In helpful episodes, participants located change first in their bodies or in the direction of attention. They noticed shoulders dropping, breathing deepening, jaws unclenching, posture becoming less contracted, or restless movement stopping. P06 linked quiet humming to a shift in writing stance:

*The breathing and humming changed my bodily state first, and then my way of thinking followed. If I had stayed tense like that, I would not have thought of writing from another angle. What music did was not to* “give me inspiration”*, but to* “open a channel”*. (P06)*

Attention then moved away from self-evaluation or a repeatedly inspected detail. P04’s concern changed from “What I have written is terrible” to “I only need to adjust the structure here”, a shift from negative self-judgement to identifying the problem (P04). P09 moved from questioning the analysis’s value to stating one claim clearly. In technical tasks, attention moved upstream: P05 reconsidered a beam’s constraint, P08 reread boundary conditions, P12 returned to approximation assumptions, and P14 checked a variable’s construction. P08 called this “clearing the default assumption so that I could find the entry point again” (P08).

Re-engagement was identified in accounts of reopening software, turning to an earlier note, entering a formula, finding textual evidence or beginning to type. P01 called music a “restart button”, while stressing that it did not write the report; work was reported to resume (P01). P07 likewise called it a buffer that made a solution discoverable, not the solution. These descriptions distinguished task return from a general claim about motivation. P02 said, “I did not become ‘more motivated’; the obstacle was removed” (P02).

Not every helpful episode required music to end before work resumed. P02 continued a predictable instrumental playlist while completing a proof, and P04, P07 and P09 retained low-salience music during writing, data handling or other familiar operations. P09 described it as an external pulse that supported continuity:

*Music acted more like a* “stable external rhythm”*. It contributed nothing to the content, but it allowed me to maintain a steady pace of output while writing, rather than stopping for a long time whenever I became stuck. (P09)*

Those working with language, demanding calculations or internal auditory imagery instead removed the music after the transition. P05’s account of falling asleep and P03’s description of extending listening to avoid work constrained Theme 2: bodily or emotional change counted as a perceived transition only when a task-relevant action was reported. Neither the action nor its quality was independently observed.

### 4.3 Perceived helpfulness depended on fit rather than genre

The unhelpful episodes showed that a successful use in one situation could reverse in another. P02 found that instrumental music compatible with mathematical reasoning interfered when chemical material had to be recited. P03’s evocative piece opened associations for literary analysis but drew attention from the exact wording of a classical text. P10 said, “The same piece of music put my mind into a state suited to making associations, but that was precisely the state I did not need when trying to memorise material” (P10). P04 and P12 found even unobtrusive, non-lyrical sound burdensome during formula- or symbol-dense work; P09 and P14 removed music for conceptually dense English reading.

No preferred genre explained these contrasts. Familiarity, predictability, intelligible lyrics, volume, emotional direction and perceptual salience entered the judgements. P01’s familiar old song accompanied a useful pause, whereas a new, catchy song left a persistent rhythm and changed anxiety into “anxiety plus distraction” (P01). P06 stopped an unfamiliar, harmonically rich song when vocal training prompted analysis. P08 removed a narratively striking soundtrack from a study playlist because its narrative movement continued to occupy attention. P12 and P14 used a minimally changing sound field intended to be ignored; P14 said, “If a piece cannot be ignored, it has failed” (P14).

Musical training altered what could be ignored. P06 attended to vocal production, P09 to instrumental arrangement, P11 to musical structure, and P15 to timbre and bodily memory. That sensitivity could support fine-grained selection or turn background use into analysis, appreciation or embodied following. P11 summarised the boundary as “how much ‘auditory blankness’ the task required”: external music could open possibilities while a direction was being sought yet compete with internally generated material during precise implementation (P11).

Fit also changed with fatigue, deadline pressure and the surrounding soundscape. P05’s familiar rock interrupted a technical loop when alert, but after sleep loss it accompanied falling asleep: “Music cannot rescue you when you are already sleepy; it only lets you fall asleep more comfortably” (P05). P03, P07 and P14 said an internal deadline countdown could outweigh listening. P02, P04, P07, P12 and P14 valued a sound barrier in noisy settings; when the room was quiet, silence could provide the desired condition. Within-person reversals, such as P10’s contrasting uses of the same piece, therefore displaced a genre-based account in Theme 3 in favour of situated fit.

### 4.4 Monitoring, stopping and choosing silence

Participants monitored bodily, attentional and behavioural signals. P01 noticed a relaxed neck; P04 stopped scrolling over the same paragraph; P06 saw words appearing on screen; P07 stopped rocking and clicked into the spreadsheet; P11 returned to the piano; and P15 felt an impulse to take up a pen. P04 also applied a ten-minute test, P09 reviewed what had been produced, and P12 compared reading speed with the usual pace in silence.

Monitoring could lead to continuation, switching or deliberate closure. P03, P06 and P13 treated a desire for another song as a possible sign that regulation had become avoidance. P03 explained, “if I still want to listen to a second one, that means I am still ‘avoiding’ the task”; wanting to stop and open the book indicated readiness to return (P03). P08 judged a background track unsuccessful if a musical passage remained in mind afterwards. P13 understood removing the headphones as “a psychological commitment” to begin serious work (P13). In contrast, P02, P04, P07 and P09 continued listening when the sound remained unobtrusive and the task progressed.

These judgements developed through trial and error into differentiated personal rules, not a shared prescription. P05 questioned dependence on a start-up ritual; P02 and P07 raised the cost of prolonged headphone use; and P11 treated “actively choosing not to listen” as a meaningful musical decision (P11). Silence, walking, reading aloud or changing the task were alternatives when the match was wrong. These cases bounded Theme 4: monitoring included recognising avoidance, dependence and discomfort, rather than simply refining continued listening. They did not demonstrate that students invariably recognised failure or successfully changed strategy.

## 5 Discussion

This study examined how university students understood self-directed music use in bounded episodes of academic stress. Four themes concerned assigning music a job, describing a transition to action, judging contextual fit and monitoring whether to continue. Figure 1 summarises their relationships as an interpretive account; the links are not tested causal effects or a sequence required in every episode.

**Figure 1:**
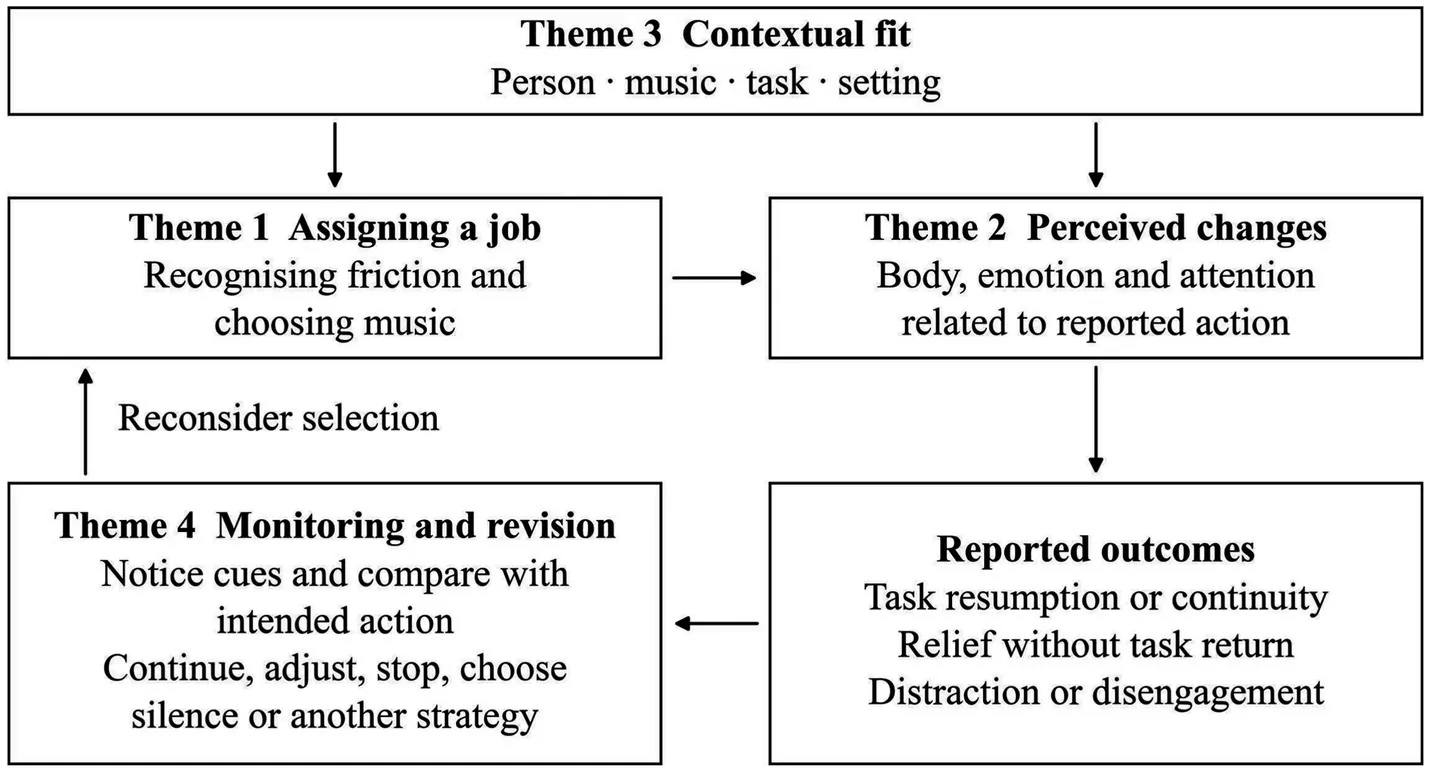
Interpretive relationships among the four themes. Arrows summarise relationships interpreted from retrospective accounts, not tested causal effects or a sequence followed in every episode. Contextual fit conditions selection and perceived consequences. Emotional change is not required for reported task return, and reported return does not establish improved learning or productivity.

### 5.1 Transition regulation and re-engagement

The central finding was that participants distinguished relief from returning to academic action. Students recognised friction—bodily tension, repetitive checking, self-criticism or environmental noise—and selected music to interrupt, shield, activate or attune themselves. This resembles the identification, selection, implementation and monitoring functions of the extended process model (Gross, 2015; McRae and Gross, 2020). The accounts add a reported task endpoint: feeling calmer alone did not count as re-engagement; students described reopening a document, revising an assumption or continuing a calculation. Task-level self-regulated learning connects these accounts to the reinvestment of attention and effort (Theobald, 2021; Zheng et al., 2023). Resuming work nevertheless says nothing by itself about accuracy, comprehension, retention or productivity.

This distinction extends work showing that students weave music into learning and wellbeing (Hu et al., 2021) and regard it as an accessible coping resource (Vidas et al., 2021, 2022). Reconstructing particular incidents exposed a step that broad reports can obscure: relief and renewed work were related but separate outcomes. Several participants denied becoming more motivated or receiving an answer; an obstacle was removed so that an existing intention could be enacted. This offers a situated reading of the association between music emotion regulation and problem-focused coping (Leipold et al., 2025). Music may prepare the ground for problem-focused action, but neither that observational evidence nor these retrospective accounts establishes causation or improved performance.

The accounts also differentiate the processes coordinated around task return. P02’s acoustic shielding primarily concerned environmental management; redirecting attention from repeated checking concerned attentional regulation; reduced agitation concerned emotion regulation; and re-examining a boundary condition concerned study-strategy adaptation. One episode could contain several processes, so overlapping codes preserved their co-occurrence without treating them as equivalent. Environmental change could be followed by task return without a reported emotional shift, while emotional relief could occur without task return. The framework therefore links these processes through an academic goal rather than assuming that emotion regulation mediates every pathway (Harley et al., 2019).

### 5.2 Dynamic person–music–task–context fit

Every participant supplied an unhelpful or counterproductive episode. This helps explain mixed background-music findings: usefulness was relational, not a stable property of a genre or playlist (de la Mora Velasco and Hirumi, 2020). Students adjusted timing, intelligible language, predictability, prominence and stopping. Although everyday choices vary across reading, writing and memorising (Goltz and Sadakata, 2021), the paired incidents show more sharply that one person’s preferred strategy can reverse across tasks.

The clearest boundary was the task’s immediate demand. Music seemed manageable during familiar execution, data handling, some reasoning or open association, yet burdensome during verbal rehearsal, precise symbolic reading, dense second-language processing or internal auditory imagery. References to a ‘phonological loop’ or ‘working-memory channel’ were participants’ explanations, not measured mechanisms. The pattern is nevertheless compatible with evidence that music can add cognitive effort under demanding conditions (Arboleda et al., 2022). It also cautions against equating subjective focus with objective gain: preferred music increased self-reported focus in a low-demand vigilance task without improving reaction-time outcomes (Kiss and Linnell, 2021).

Musical salience was also conditional. Familiarity and predictability often let sound recede, whereas catchy, novel or professionally interesting material captured attention. Yet some students deliberately chose emotionally congruent music for creative association, or energising music for activation. Musical training made a recording more informational, sharpening selection whilst increasing the chance of analysis or embodied following. Fatigue, deadline intensity, room noise and available silence shifted the threshold again. These within-person reversals complement heterogeneous experimental stress-recovery findings and survey evidence of varied self-selected listening choices (Adiasto et al., 2022, 2023). Asking which music ‘works’ is therefore incomplete without specifying the person, goal, task phase and setting.

The Chinese university setting gives these practices a concrete social and educational context. P02 described peer comparison in a class messaging group, while P04 described silent headphones as a signal against interruption in shared accommodation. P01 worried that headphone use might be read as not studying, while P03 and P10 contrasted associative reading with exact textual memorisation. Such accounts suggest that peer visibility, shared study space and assessment demands shaped both the perceived legitimacy of listening and the need to protect attention. They resonate with intertwined learning and well-being uses reported in Chinese universities (Hu et al., 2021). These features are not uniquely Chinese, and the study did not compare national groups or measure cultural values; they identify contextual conditions for transferability rather than evidence of a uniform Chinese learning culture.

### 5.3 Monitoring the regulator

The findings develop the monitoring stage of process models by showing the small tests through which students evaluated music (Gross, 2015; McRae and Gross, 2020). They watched for a relaxed jaw, orderly notes, restored typing or a desire to reopen the task, and checked what had been produced after several minutes. They then continued, altered the sound, stopped or chose silence. Stopping was not simply failure; it could complete a planned transition or protect a more demanding task phase.

Monitoring also operated across episodes. P04’s ten-minute check, P09’s review of written output and P12’s comparison with reading in silence suggest a metacognitive cycle of noticing a discrepancy, comparing current action with a personal reference point, and revising a listening rule. P03’s distinction between wanting another song and wanting to open the book illustrates self-observation of avoidance rather than mood alone. Trial and error may thus build conditional knowledge about when to retain, alter or stop music. These are interpretations of narrated practices, not measured differences in metacognitive ability. More elaborate accounts may reflect experience, task knowledge or interview articulacy; the data cannot establish that those students monitored more accurately or had greater cognitive capacity.

The failure cases also set a boundary around this interpretation. P02 repeatedly preferred silence for recitation, P10 excluded music from precise memorisation, and P11 protected internal auditory space during detailed musical work. P12 and P14 also described narrowing listening after repeated mismatches. These are recurring task-specific failures rather than evidence of a group for whom music was consistently ineffective across situations. All 15 participants supplied both helpful and unhelpful accounts; the study therefore cannot characterise people who abandoned music after repeated failure. Stopping may express adaptive monitoring, but it may also follow an unsuccessful attempt that has already consumed time. Associations between some music-regulation patterns and emotional symptoms reinforce the need to examine these possibilities without diagnosing participants (Morgan and Marroquín, 2024; Tan et al., 2024).

### 5.4 Implications for higher education

The findings offer questions for student-support discussions, not evidence-based intervention recommendations. Everyday listening here was neither a standardised intervention nor clinical music therapy. Evidence from controlled music interventions cannot establish the effectiveness of guidance derived from these interviews because the activities, populations and outcomes differ (de Witte et al., 2020; Peters et al., 2024). Any educational use of the proposed monitoring questions would require co-design and prospective evaluation.

Possible discussion questions include what a student wants listening to change, what the next task requires, and what would count as a reason to stop. The accounts suggest examining reported academic action alongside mood, and considering silence or another strategy when sound competes with language, increases drowsiness or extends avoidance. These questions translate the dynamic logic of regulation into a candidate resource for evaluation, without establishing an optimal playlist, trial duration or sequence of actions (Gross, 2015; Adiasto et al., 2022, 2023).

Participants’ accounts also invite consultation about access to quiet space and the meanings attached to headphones. Headphones sometimes marked an effort to manage noise or avoid interruption; their presence alone did not establish disengagement. Student-centred consultation could explore these experiences alongside non-users’ preferences, given the cognitive, emotional and social functions of university music use (Dingle et al., 2021; Hu et al., 2021). Such consultation would not substitute music for professional support when distress persists.

Algorithmic curation raises additional design questions. Qualitative evidence connects perceived stress recovery with trust and emotional autonomy while identifying privacy concerns (Zheng et al., 2026). User control and the option of silence are therefore relevant topics for any future co-design work, although this study evaluated neither an app nor an institutional playlist.

### 5.5 Limitations and future research

Several limitations bound these interpretations. The purposive sample comprised 15 undergraduates from two public universities in eastern China who had intentionally used music during academic stress. Maximum variation broadened contrasts within existing users, not across the undergraduate population. Students who rarely listen, favour other coping strategies or have abandoned music may identify different triggers and pathways, or none involving music. The accounts cannot establish that those students would benefit from adopting it. Exclusion of students receiving clinical treatment further limits transferability. Counsellor-assisted recruitment may have favoured volunteers comfortable discussing stress with researchers; separation from assessment and confidential handling reduced some power concerns but cannot establish that recruitment relationships had no influence.

Critical-incident interviews rely on memory and retrospective meaning-making. Participants may have selected unusually memorable episodes, compressed timing or reconstructed a coherent progression because the guide asked about events before, during and after listening. Goals described in retrospect may not have been explicit at the time, and a later insight may have been attributed to music after the task resumed. The proposed ordering is therefore an account of remembered experience, not a verified within-episode mechanism. Listening also accompanied breathing, walking, drinking water or resting the eyes, so its contribution cannot be separated from the micro-break. No listening logs, task traces, physiological measures or performance tests corroborated the accounts. Consequently, reported task return establishes neither objective improvement nor that music was necessary for it. Translation from Mandarin, despite bilingual checking, added another interpretive layer.

The interpretivist analysis was shaped by the researchers’ theoretical interests and the lead researcher’s listening practices. Reflexive records, negative-case analysis and critical-peer dialogue made those influences visible but did not remove them. The result is a contextual interpretation, not an effect estimate or reproducible code-frequency model.

Transferability beyond these settings depends on whether relevant conditions are shared. Access to quiet space, norms around headphones, language and assessment demands, and acceptable ways of taking a study break may alter both music selection and its interpretation. Cross-cultural use of the model should therefore examine these conditions rather than assume that either the four themes or their ordering will recur unchanged.

Future designs could test specific propositions generated by these accounts. Event-contingent diaries or ecological momentary assessment could record the immediate barrier, regulatory goal, listening choice and next task action close to their occurrence, testing whether reduced tension is sometimes followed by relief without resumption. Consented task traces could distinguish self-reported readiness from latency to restart and sustained activity; comprehension, accuracy or retention measures would be needed to assess learning outcomes. Within-person comparisons of music, silence and a matched non-musical break could examine whether verbal task demands and musical salience alter these outcomes while separating listening from rest. Repeated observations could test whether monitoring rules become better calibrated or remain retrospective explanations. Recruiting rare users, former users and students reporting recurrent failure, alongside samples across cultural and institutional settings, would examine when the proposed pathways are absent or take different forms.

## 6 Conclusion

This study traced how 15 existing music users interpreted intentional listening within academic-stress episodes and related it to what they reported doing next. Students assigned music particular jobs, including interrupting repetitive checking, managing noise, adjusting arousal and preparing for a task. Some described bodily or attentional change followed by renewed action; others described distraction, sleepiness, avoidance or competition with demanding academic processing. Helpfulness was understood through changing person–music–task–context fit and through decisions to retain, adjust or stop listening. The contribution is an episode-based account that distinguishes emotional relief from perceived task return and differentiates the processes students coordinated around that goal. These retrospective accounts establish neither causal effects nor improved learning or productivity, and do not support prescribing music to students who do not already use it. They offer propositions for prospective research and questions for co-designed student-support resources whose usefulness remains to be evaluated.

## Data Availability

The original contributions presented in the study are included in the article/Supplementary material, further inquiries can be directed to the corresponding author.
