# Supplementary material for: Music use under academic stress: university students’ experiences of emotion regulation and learning re-engagement
Source: Front Psychol. 2026 Oct 26;17:1989359. doi: 10.3389/fpsyg.2026.1989359 (PMC13745500; doi:10.3389/fpsyg.2026.1989359)
Supplement: Supplementary file 1 [file Supplementary_file_1.docx]

# **Appendix A. Semi-structured Interview Protocol**

## **Core Interview Questions**

## **A. Warm-up and academic context**

**1. When you think about academic stress in your current university life, what situations tend to create it? [RQ1]**

**Optional probes:** Examinations, deadlines, difficult tasks, feedback, workload, group work, competing responsibilities; what makes the situation stressful rather than merely busy?

**2. What place, if any, does music usually have in your studying or in dealing with academic pressure? [RQ1]**

**Optional probes:** Listening before, during, or after studying; singing or playing; usual devices, places, and routines. Do not assume regular use.

## **B. A recent music-use episode**

**3. Please take me to one recent, specific occasion when academic work felt stressful and you intentionally turned to music. What was happening? [RQ1]**

**Optional probes:** Task and setting; people present; timing; immediate trigger; thoughts, emotions, bodily sensations, and behaviour before the music.

**4. At that moment, what did you want the music to change—or help you keep the same? [RQ1]**

**Optional probes:** Calm down, gain energy, accept or understand feelings, stop worrying, feel accompanied, prepare to focus; was the goal clear at the time?

**5. How did you decide to use music rather than another response, and how did you choose what to hear or do? [RQ1; RQ3]**

**Optional probes:** Song/playlist/genre without quoting lyrics; familiarity, tempo, language, memories, recommendations, shuffle; headphones/speakers; listening, singing, or playing.

**6. Please walk me through what happened while the music was present and immediately afterwards. [RQ2]**

**Optional probes:** Changes over time; attention to music versus the task; actions such as moving, resting, planning, or starting work; any moment when the effect shifted.

**7. What, if anything, changed in your emotions, bodily state, thoughts, attention, or motivation? How did you know? [RQ2]**

**Optional probes:** Intensity and direction of change; relief versus understanding; distraction versus refocusing; energising versus settling; no change or mixed effects.

**8. What was the next academic action you took, and what role did the music have in it, if any? [RQ2]**

**Optional probes:** Started, returned to, continued, postponed, switched, or abandoned the task; time to return; attention, effort, persistence, planning; distinguish feeling ready from actually acting.

**9. Did the music continue once you were working? How did you decide whether to keep it, change it, or turn it off? [RQ2; RQ3]**

**Optional probes:** Monitoring; volume; lyrics; task difficulty; switching playlists or to silence; signs that the music was helping or competing for attention.

## **C. When music did not help**

**10. Now think of a time when music did not help with academic stress, helped only briefly, or made studying harder. What happened? [RQ2; RQ3]**

**Optional probes:** Delay, avoidance, rumination, intensified emotion, over-absorption, distraction, fatigue, mismatch with task or mood; it is acceptable if no example comes to mind.

**11. How did that episode differ from the earlier one, and what did you do next? [RQ2; RQ3]**

**Optional probes:** Regulatory goal, initial stress/emotion, music choice, timing, setting, task, other people, switching strategy, and eventual learning action.

## **D. Conditions, learning, and adaptation**

**12. What personal factors shape whether music helps you return to learning? [RQ3]**

**Optional probes:** Current mood and stress intensity; preferences; habits; music training; need for stimulation; past associations; individual differences.

**13. Which features of the music or the way you use it make a difference? [RQ3]**

**Optional probes:** Familiarity, lyrics/language, tempo, intensity, genre, volume, playlist control, duration, headphones, active versus background listening.

**14. Which features of the academic task or surrounding situation make a difference? [RQ3]**

**Optional probes:** Reading/writing versus routine work; novelty and difficulty; deadline proximity; noise; place; social presence; time of day.

**15. How have you learnt to judge whether music is working, and have you changed your approach over time? [RQ3]**

**Optional probes:** Signals monitored; trial and error; rules or playlists developed; stopping points; alternative strategies; occasions when silence is chosen.

## **E. Meaning and close**

**16. Looking across these experiences, what does using music under academic stress mean to you? [RQ1–RQ3]**

**Optional probes:** Resource, routine, reward, companion, avoidance, identity, boundary between coping and learning; any tensions or contradictions.

**17. What would you want university educators or student-support staff to understand about music and returning to study? [RQ2; RQ3]**

**Optional probes:** Useful support without prescribing one solution; differences between students; possible risks or limits.

**18. Is there anything important that I have not asked, or anything you would like to clarify? [All RQs]**

**Optional probes:** Invite additions. Summarise the main account and ask whether it is fair.

# **Appendix B. Final Coding Framework and Analytic Trail**

### **B1. Purpose, epistemological position and status of the framework**

This appendix documents how the final interpretation was developed from the 15 de-identified Mandarin interview transcripts (P01–P15). The study used reflexive thematic analysis (RTA) within an interpretivist, contextualist orientation. Participants’ accounts were treated as situated interpretations of remembered events, co-produced in the interview, rather than as transparent measurements of a single objective mechanism. The analysis therefore sought patterns of meaning in how students understood intentional music use around episodes of academic stress and learning re-engagement. It did not test whether music objectively improved emotion, cognition, task performance or attainment.

The framework below is a final, evolving analytic map. It is not an a priori codebook, a set of instructions for a second coder, or an inter-rater reliability instrument. Early labels were provisional and were repeatedly split, merged, renamed and repositioned as whole transcripts, helpful episodes, mixed experiences and counterproductive incidents were compared. A single extract could carry several codes because bodily, affective, attentional, cognitive, environmental and behavioural meanings often co-occurred. Code and participant coverage helped the researchers inspect the range and boundaries of an interpretation; neither frequency nor agreement was used to determine thematic importance.

Initial coding was primarily experiential and semantic, staying close to participants’ expressed meanings and episode sequences. The extended process model of emotion regulation and the task-level self-regulated learning (SRL) lens informed later interpretation as sensitising resources. They helped the researchers ask how a need was identified, how a strategy was selected and implemented, how its effects were monitored, and whether a student resumed or sustained a specific academic action. They did not supply fixed coding categories or dictate the final themes. The final themes were organised through participants’ accounts of friction, assigned purpose, transition, fit and monitoring, rather than being named after theoretical stages.

NVivo Release 14 (Lumivero), as reported in the main Methods section, was used to organise transcripts, linked extracts, evolving code labels and analytic memos. It did not code the data automatically, calculate themes or make interpretive decisions. No kappa coefficient, percentage agreement or consensus-coding threshold was used. Critical-friend dialogue described in the Methods was used to question assumptions, test alternative readings and sharpen thematic boundaries, not to certify one objectively correct coding solution.

### **B2. Recursive analytic pathway**

The stages below are presented sequentially for audit clarity; in practice, movement between them was recursive.

| **Analytic movement** | **What was done** | **Analytic trace retained** | **Reflexive question used** |
| --- | --- | --- | --- |
| 1. Familiarisation in Mandarin | Each complete transcript was read and reread. Notes attended to the stressful situation, the purpose assigned to music, the timing of listening, perceived changes, the next academic action, and any unhelpful or contrary episode. | Transcript-level notes and brief episode summaries. | What is the participant claiming music was for in this episode, and what remains outside that claim? |
| 2. Close, provisional coding | Meaning units were coded in participants’ own terms where possible. Coding was mainly semantic and experiential, although metaphors such as ‘restart button’, ‘wall’, ‘corridor’ and ‘operating environment’ were retained where they condensed a wider meaning. Several codes could be attached to one extract. | Linked extracts, evolving labels and reflexive memos. | Am I coding a reported experience, or importing a preferred explanation of how music should work? |
| 3. Episode mapping | Each focal account was mapped as a temporal sequence: academic friction → regulatory goal → music choice/use → bodily, affective, attentional or cognitive change → next academic action → monitoring. Helpful and counterproductive episodes were mapped within the same participant wherever available. | Fifteen within-case maps and episode-valence notes. | Does the account show task re-engagement, or only a temporary improvement in mood? |
| 4. Consolidating code families | Related provisional labels were clustered into broader families concerning friction, assigned purpose, placement/selection, state transition, attentional and problem reorientation, behavioural re-engagement, conditions of fit, and monitoring. Families remained revisable and overlapping. | Successive code-family tables and memos documenting merges and distinctions. | Which labels describe the same underlying analytic idea, and which apparently similar labels perform different functions? |
| 5. Developing candidate themes | Candidate patterns were organised around central concepts rather than topics. Comparisons were made across disciplines, levels of musical experience, pre-task and concurrent listening, and reported helpful, mixed and counterproductive outcomes. | Candidate thematic maps and cross-case matrices. | What shared story links these extracts, and what would be lost if the pattern were reduced to ‘music is relaxing’? |
| 6. Interrogating boundaries and disconfirming cases | Candidate themes were reviewed against coded extracts and complete transcripts. Within-person reversals were prioritised because they showed how the same listener or music could produce a different result under altered task demands. Minority and boundary cases were retained when they changed the interpretation. | Negative-case memo, revised thematic maps and decision log in Section B6. | What evidence complicates or reverses this interpretation? Does the theme explain the contrary event without explaining it away? |
| 7. Defining, naming and writing | Four final themes were defined by distinct central organising concepts. Their relationship to each RQ and the two sensitising lenses was articulated only after the data-led thematic structure was stable. Analytic writing prompted further refinement. | Final theme definitions, subthemes, cross-case matrix and quotation audit (Appendix C). | Is each theme internally coherent, distinguishable from the others and capable of carrying an interpretive claim rather than merely listing a topic? |
| 8. Translation for reporting | Coding and theme development remained in Mandarin. Final theme labels and selected quotations were rendered in English after the structure stabilised and checked for conceptual and affective equivalence as described in the Methods. | Bilingual quotation chain in Appendix C, including source locators and translation notes. | Does the English preserve the participant’s distinction, force and metaphor without becoming more polished or causal than the Mandarin? |

An exact count of ‘initial codes’ is not reported because code labels were working analytic objects rather than fixed units: they were renamed, combined, divided and applied at different levels during recursive analysis. Presenting a single number would imply a stability that the analytic process did not have.

### **B3. Consolidated code families**

The following families summarise the final analytic vocabulary. The example codes are illustrative rather than exhaustive or mechanically reproducible inclusion rules.

| **Code family** | **Analytic focus** | **Illustrative final codes and distinctions** | **Principal RQ contribution** |
| --- | --- | --- | --- |
| CF1. Academic friction and regulation-need identification | What made the event more than ordinary busyness, and how the learner recognised that the current approach was no longer working. | Deadline compression; technical or epistemic blockage; repeated checking; blank-page or transition threshold; evaluative self-criticism; outcome uncertainty; environmental acoustic intrusion; fatigue or low cognitive reserve; bodily signs of being stuck. | RQ1 |
| CF2. The ‘job’ assigned to music | The particular change sought from music at that moment. | Interrupt or reset an unproductive loop; create psychological distance; construct a sonic boundary; stabilise attention or output rhythm; increase activation; soften overactivation; attune emotion or perception to a text/creative problem; mark entry into study. | RQ1; RQ3 |
| CF3. Music placement and selection | How music was positioned in relation to the task and configured for the desired job. | Low-friction/immediate access; ritual selection; bounded pre-task or mid-task interval; concurrent background; familiar/predictable choice; lyric or voice management; level of musical information; tempo and rhythmic stability; affective congruence; volume; headphones versus speakers; active humming or playing as a boundary case. | RQ1; RQ2; RQ3 |
| CF4. Embodied and affective transition | How participants noticed that their state had become more or less workable. | Breathing deepened or slowed; shoulders, jaw or back released; posture shifted; restless movement stopped; bodily warmth or activation changed; irritation softened; calm alertness, confidence or felt possibility returned; over-soothing, sleepiness or emotional immersion. | RQ2 |
| CF5. Attentional and problem reorientation | How the direction, breadth or level of attention changed, and how existing task material became differently accessible. | Detaching from perseveration; masking external distraction; widening from local detail to whole structure; narrowing scattered possibilities; evaluation-to-execution shift; moving from output to input or calculation to premise; retrieving an overlooked note, memory or textual detail; external pacing; attention capture, semantic competition and stylistic contamination. | RQ2; RQ3 |
| CF6. Proximal learning re-engagement | The observable task action that followed, without treating reported calm as an educational outcome in itself. | Reopening software/document; rereading the task; picking up a pen; inspecting an earlier file; writing a core claim; locating evidence; revising a premise; recalculating; completing a graph, paragraph, section or further exercises; sustained typing or task effort. | RQ2 |
| CF7. Relational fit and reversal conditions | Why a strategy worked in one episode but was ineffective or harmful in another. | Task processing demand (association, execution, reading, language decoding, symbol discrimination, memorisation, internal auditory imagery); music salience (lyrics, novelty, complexity, narrative pull, stability, timbre, volume); personal state (sleep, result anxiety, expertise, emotional associations, prior listening); context (noise, privacy, device, light, time, weather); semantic interference; attention residue; drowsiness; affective mismatch; delay. | RQ2; RQ3 |
| CF8. Monitoring, modification and costs | How students judged and updated the strategy within and across episodes. | Bodily/action cue; output/time test; desire to return versus desire for another song; lingering melody; continue, switch or stop; choose silence, earplugs, movement, reading aloud, water or another tool; develop task-specific playlists; hearing comfort, auditory fatigue or ritual dependence. | RQ3; RQ2 |

### **B4. Final thematic framework**

### **Theme 1. Giving music a job at moments of friction**

**Central organising concept.** Intentional music use began when students recognised a particular point of friction and assigned music a bounded regulatory or environmental task. Music was not selected simply because it was liked or assumed to be relaxing. It was asked to interrupt a stuck loop, construct a workable soundscape, stabilise pace, increase activation, or attune the learner to a text or creative problem.

| **Element** | **Final specification** |
| --- | --- |
| Subtheme 1.1. From ‘busy’ to ‘stuck’ | The need to regulate became visible at a concrete loss of traction: a repeated error, uncertain premise, anomalous result, fragmented argument, blank opening, frozen creative direction or inability to start. Emotional and bodily cues—irritation, heaviness, self-doubt, shallow breathing, muscular rigidity and repetitive screen behaviour—helped students identify the impasse. |
| Subtheme 1.2. A restart, sonic enclosure or state-tuning device | Students gave music different jobs. Reset/interruption was prominent for P01, P05, P07, P08, P12 and P14. Sound masking or output stabilisation was prominent for P02, P04, P07, P09, P12 and P14. Affective, perceptual or creative attunement was prominent for P03, P06, P10, P11 and P15, while P05 sought activation and P13 sought convergence on one line of argument. |
| Supporting evidence | P01 described a difficult-task ‘ritual’ and later a ‘restart button’ ([P0009], [P0032]); P02 wanted music to become a sound-blocking ‘wall’ ([P0072], [P0098]); P05 stated, ‘I do not need to be soothed; I need to be activated’ ([P0220]); P09 used steady music as an external writing rhythm ([P0434]–[P0446]); P10 used a pre-writing corridor into the emotional texture of a literary text ([P0486]–[P0498]); P12 and P14 described low-information sound as a way to interrupt internal circling rather than improve mood ([P0591]–[P0599], [P0695]–[P0703]). |
| Contrasting and boundary evidence | P02, P12 and P14 explicitly downplayed an emotion-regulation account and primarily described environmental or attentional management. P11’s listening sometimes formed part of disciplinary creative work, not merely coping. Timing also varied: P01, P03, P05, P06, P10, P13 and P14 mainly used a bounded interval; P02, P04, P07 and P09 often used concurrent background sound; P08, P11, P12 and P15 shifted with task phase. These variations prevented ‘pre-study relaxation’ from becoming the theme. |
| RQ link | Primary: RQ1 (stress situation and regulatory goal). Secondary: RQ3 (situational and person-specific selection). |
| Sensitising theoretical link | The extended process model sensitised attention to identification and selection: students detected a need and selected a strategy for a particular goal. Task-level SRL helped interpret the friction as a problem of initiating, protecting or restoring task engagement. The theme was not derived from those theoretical stages. |

### **Theme 2. Music made the transition; students did the academic work**

**Central organising concept.** In helpful accounts, music did not provide academic content or complete the task. Participants described it as structuring a transition in which bodily or affective strain shifted, attention was redirected, an existing problem could be represented differently, and a concrete academic action followed. Re-engagement was identified through what the student next did, not through feeling better alone.

| **Element** | **Final specification** |
| --- | --- |
| Subtheme 2.1. A bodily or affective loosening made movement possible | Across the sample, participants noticed changes in breathing, jaw or shoulder tension, sitting position, stillness, warmth or activation. These were experiential signs of a changed state, not physiological measurements. The desired state was often calm alertness or workable energy rather than happiness. |
| Subtheme 2.2. Attention moved from self-evaluation or local entanglement to a tractable representation | Some accounts widened attention from a repeated local error to an upstream condition (P05, P07, P08, P12, P14). Others opened an associative or expressive route (P03, P06, P10, P11, P15). P09 and P13 narrowed scattered material into an executable line. Several participants described a shift from judging themselves to locating the next problem. |
| Subtheme 2.3. The next action marked re-engagement | Re-engagement became visible when a participant reopened a file, reread the question, wrote a core sentence, searched for evidence, changed an assumption, recalculated, returned to an instrument or continued producing text. Every helpful focal episode included a specific subsequent academic action; reported calm without task action was not treated as re-engagement. |
| Supporting evidence | P02 said motivation did not increase; rather, an obstacle was removed and orderly proof-writing resumed ([P0080]). P04 shifted from ‘my writing is terrible’ to locating a structural problem and wrote for a further period ([P0184]–[P0186]). P06 linked breathing and humming to a later change in writing perspective and natural typing ([P0284]–[P0290]). P08 shifted from ‘Where did I calculate incorrectly?’ to ‘Where did I think incorrectly?’ and reread the boundary conditions ([P0388]–[P0394]). P11’s awareness was ‘opened’, not ‘told’, before renewed composition ([P0547]–[P0551]). P15 described bodily regulation preceding the recovery and reorganisation of earlier material ([P0755]–[P0759]). |
| Contrasting and boundary evidence | Music was intertwined with closing the eyes, leaning back, drinking water, walking, looking outside, screen withdrawal, humming or rereading. The accounts therefore support an experienced transition, not the causal isolation of sound. P01, P02 and P07 explicitly indicated that motivation itself had not necessarily risen. P09 sustained output while music continued; P06, P08 and P10 needed music to stop before high-load work. The common element was movement into task action, not a universal process direction or listening duration. |
| RQ link | Primary: RQ2 (perceived emotional, attentional and cognitive processes, followed by learning action). |
| Sensitising theoretical link | The emotion-regulation lens sensitised attention to what happened during and after strategy implementation. Task-level SRL defined the educational consequence proximally: restored attention, effort, persistence or strategy-directed action on the focal task. The data do not support a claim of improved grades, learning quality or objective cognitive capacity. |

### **Theme 3. Fit, not genre, determined whether music helped**

**Central organising concept.** Whether music supported or hindered re-engagement depended on a dynamic relation among the desired state, the academic processing demand, the salience of the sound, the learner’s current capacity and experience, and the surrounding environment. No genre or feature was intrinsically helpful. Every participant reported an unhelpful, mixed or counterproductive episode, often reversing a strategy that had worked elsewhere.

| **Element** | **Final specification** |
| --- | --- |
| Subtheme 3.1. Task processing demands drew the clearest boundary | Language decoding, subvocal rehearsal, precise memorisation, symbol discrimination, categorical placement and internal auditory imagery were commonly described as competing with music. Routine operations and some familiar calculations could tolerate low-salience sound. Associative, expressive or creative work sometimes benefited from brief attunement, but precise execution within the same discipline could require silence. |
| Subtheme 3.2. Musical salience had to serve the goal | Familiarity, predictability, stable rhythm, low volume and low linguistic or structural information frequently allowed sound to recede. Yet low salience was not universally desirable: P03 and P10 chose affectively congruent material, P05 sought activating music, and P11 deliberately used complex contrasting work to widen creative perception. The higher-order question was whether salience served the assigned job. Novelty, intelligible lyrics, technical interest, narrative pull, abrupt change or embodied familiarity could become distractors. |
| Subtheme 3.3. Personal reserve, training and setting changed the threshold | Fatigue, poor sleep, auditory depletion and intense outcome anxiety reduced the available capacity for additional sound. Musical training increased both strategic sensitivity and involuntary analysis or bodily following (especially P06, P09, P11 and P15). Environmental noise increased the value of masking; an already adequate quiet environment often made music unnecessary. Devices, privacy, time, natural light and weather also entered participants’ explanations. |
| Supporting evidence | Paired incidents occurred throughout the dataset. P03 described the same affective music as useful for literary association but disruptive for exact verbal recall ([P0128]–[P0140]). P05 found familiar music activating when alert but sleep-inducing when exhausted ([P0230]–[P0244]). P07 tolerated familiar lyrical music during routine report production but not formula memorisation ([P0344]–[P0348]). P08 showed that even lyric-free music could distract when unfamiliar and narratively salient ([P0398]–[P0404]). P11 needed external music for direction-finding but ‘auditory blankness’ for precise orchestration ([P0555]–[P0571]). P15’s embodied violin familiarity loosened expressive writing yet disrupted categorical art-history memory ([P0753]–[P0765]). |
| Contrasting and negative-case evidence | The data contradicted simple prescriptions. Instrumental music was not uniformly unobtrusive (P08, P11, P12, P14); lyrics were not uniformly harmful (P05 used familiar lyrics for activation, and P07 tolerated them during routine work); familiarity did not always protect attention (P05 became sleepy and P15’s body followed a familiar performance); creative work did not have a single requirement (P09 warned that steady rhythm could over-narrow highly associative thinking). P12 and P14 sometimes described useful sound as nearly ‘not music’, keeping acoustic masking analytically distinct from affective attunement. |
| RQ link | Primary: RQ3 (person, music, task and situation conditions). Secondary: RQ2 (processes through which mismatch prolonged disengagement). |
| Sensitising theoretical link | The extended process model supported interpretation of strategy–goal–situation fit and the monitoring of implementation consequences. SRL framed music as a task-contingent strategy whose usefulness depends on the processing requirements of the next academic action. Participants’ references to ‘working memory’ or ‘language channels’ remain their own explanations, not neuroscientific evidence. |

### **Theme 4. Using music well also meant knowing when to stop**

**Central organising concept.** Students were not passive recipients of music. They monitored bodily, attentional and productivity cues; continued, changed or ended listening; selected silence or another strategy; and refined personal rules through repeated episodes. Effective use therefore included regulating the regulator. Stopping music was often an agentic sign of task entry rather than evidence that music had failed.

| **Element** | **Final specification** |
| --- | --- |
| Subtheme 4.1. Students used proximal cues to judge fit | Monitoring cues included relaxed shoulders or jaw, an impulse to move the hand towards the task, organised written steps, reading time, amount produced, repeated rereading, whether the melody remained mentally foregrounded, whether a task-related image appeared, and whether the learner wanted to return or listen to another song. These markers were idiosyncratic but action-oriented. |
| Subtheme 4.2. Stopping, continuing and silence were active choices | P01, P03, P05, P06, P08, P10, P11, P12, P13, P14 and P15 commonly ended music before a high-load phase. P02, P04, P07 and P09 sometimes continued compatible low-salience background sound. Across cases, students also moved to silence, earplugs, reading aloud, walking, water, note cards or another strategy when listening did not fit. |
| Subtheme 4.3. Trial and error produced increasingly specific rules—and revealed costs | Participants described moving from indiscriminate or habitual listening towards task-specific playlists, time limits and stop tests. The same reflection exposed risks: another song could become avoidance (P03, P06, P13); extended headphone use could create hearing discomfort or concern (P02, P07); ritual dependence could reduce flexibility (P05); and trained listeners could experience auditory fatigue (P11). |
| Supporting evidence | P01 used an early ‘three-minute’ test ([P0046]); P04 checked recall after ten minutes ([P0200]); P06 distinguished an urge to write from an urge to find more songs ([P0304]); P07 and P09 judged the pairing through concrete output ([P0356], [P0460]); P08 treated lingering awareness of the music as failed backgrounding and headphone removal as a start signal ([P0408], [P0414]); P13 called removing the headphones a commitment to work ([P0675]); P15 distinguished an impulse to pick up a pen from passive immersion ([P0773]). P11 explicitly described ‘actively choosing not to listen’ as a musical decision ([P0571]). |
| Contrasting and boundary evidence | Stopping was not a universal rule. For P02, P04, P07 and P09, continuing low-salience music could sustain noise control or routine output. Conversely, a bounded ritual could become procrastination if it did not end. Active humming or playing (P06, P09 and P15) may involve bodily feedback beyond listening and was retained as a scope-extending boundary rather than folded uncritically into passive music use. |
| RQ link | Primary: RQ3 (conditions and strategy adjustment). Secondary: RQ2 (how monitoring shaped resumption or continued disengagement). |
| Sensitising theoretical link | Monitoring and modification connect naturally to the extended process model. Within task-level SRL, the same accounts show metacognitive monitoring, strategy adaptation and protection of attention. The theme extends both lenses by showing that withdrawal of the original strategy—choosing silence—can be the successful regulatory move. |

### **B5. Participant-by-theme cross-case matrix**

The matrix summarises the **type** of evidence used to test the four themes. It is not a prevalence table. Every participant provided a helpful focal episode and an unhelpful, mixed or counterproductive comparison, but the mechanism and temporal position differed. A rare boundary case could alter a theme even if it appeared in only one or two interviews.

| **Code** | **Themes 1–2: assigned job and reported transition** | **Themes 3–4: reversal/constraint, monitoring and timing** |
| --- | --- | --- |
| P01 | **T1:** Reset and difficult-task entry ritual. **T2:** Bodily settling and attentional release preceded error detection and resumed work. | **T3:** A novel, catchy song left rhythmic residue and added distraction to anxiety. **T4:** Three-minute/one-song test; switch or stop when the state does not change. **Timing:** Mainly bounded; stopped for demanding work. |
| P02 | **T1:** Sonic boundary and noise management. **T2:** Acoustic masking and calmer bodily state accompanied more orderly proof steps and completion. | **T3:** Background piano interfered with verbal rehearsal; high-volume/headphone use carried hearing concern. **T4:** Half-hour output check; music removed for verbal-memory tasks. **Timing:** Mainly concurrent, but conditional. |
| P03 | **T1:** Affective attunement and pre-writing threshold. **T2:** Emotional imagery opened an analytic angle, followed by outline and writing. | **T3:** The same piece that supported literary association disrupted exact memorisation. **T4:** Desire for another song indicated delay; self-ended listening indicated readiness. **Timing:** Bounded; stopped before writing. |
| P04 | **T1:** Stable ‘sound carpet’ for noise and self-critical thought. **T2:** Attention widened from sentences to report structure and sustained writing followed. | **T3:** The same lo-fi strategy slowed formula and statistical-output reading. **T4:** Ten-minute recall check; switch to silence/earplugs for precision. **Timing:** Concurrent for compatible synthesis; stopped when mismatched. |
| P05 | **T1:** Activation and interruption of repeated checking. **T2:** Movement, bodily reset and renewed reading of constraints preceded recalculation. | **T3:** Familiar music became a sleep cue when cognitive reserve was low. **T4:** ‘More willing to study’ versus ‘less willing to move’; awareness of ritual dependence. **Timing:** Bounded; stopped before calculation. |
| P06 | **T1:** Emotional warmth, embodied reset and narrative attunement. **T2:** Humming and breathing preceded broader explanatory logic and renewed typing. | **T3:** Complex, unfamiliar or technically salient music captured the trained voice listener. **T4:** Desire to return to the desk versus desire for more music; active stopping. **Timing:** Bounded; stopped before language generation. |
| P07 | **T1:** Sonic shield plus a brief reset. **T2:** Reduced restlessness and fresh attention made an overlooked data note visible; report work resumed. | **T3:** Lyrics competed with formula rehearsal; prolonged headphone use had a reported cost. **T4:** Word/output test and task categories; music removed for memory. **Timing:** Concurrent during compatible operations; otherwise stopped. |
| P08 | **T1:** Gentle disconnection from detail fixation. **T2:** Slower breathing and attentional reset preceded rereading of a boundary assumption and successful derivation. | **T3:** A novel, strongly narrative instrumental piece remained foregrounded during conceptual reading. **T4:** Lingering musical memory signalled failure; removing headphones signalled entry. **Timing:** Phase-sensitive; often bounded before high-load work. |
| P09 | **T1:** Stable focus, output rhythm and working soundscape. **T2:** External pacing accompanied a shift from self-evaluation to execution and sustained writing. | **T3:** The same playlist hindered coherent reading of an English-language article. **T4:** Concrete output and repeated-rereading test; pause/switch for dense checking. **Timing:** Concurrent for compatible output; conditional stopping. |
| P10 | **T1:** Affective attunement and ritual entry into literary work. **T2:** Receptive calm allowed an existing textual detail to surface, followed by searching and writing. | **T3:** A diffuse associative state blurred literary-theory classification. **T4:** Task-related imagery signalled fit; music stopped before language production. **Timing:** Bounded; stopped before writing. |
| P11 | **T1:** Perceptual opening for creative direction. **T2:** Comparative listening shifted the problem from harmony to rhythm; composition resumed. | **T3:** External music contaminated internal auditory imagery during precise orchestration. **T4:** Pencil/production pace; deliberate ‘auditory blankness’. **Timing:** Bounded and strongly phase-specific. |
| P12 | **T1:** Interruption of internal indecision and low-content noise masking. **T2:** A contentless interval weakened oscillation; rereading the premise led to solution. | **T3:** Even ambient sound reduced precision during symbol-dense conceptual reading. **T4:** Paragraph-time and action-direction cues; progressively narrower audio rules. **Timing:** Mainly bounded; minimal background only for low-load work. |
| P13 | **T1:** Ritual boundary and convergence on one central claim. **T2:** Attention moved from ‘how to write’ to ‘what to say’, followed by synthesis and sustained writing. | **T3:** Emotionally soft, gripping music produced a task-incongruent presentation state. **T4:** Urge for another song versus deliberate headphone removal. **Timing:** Bounded and self-ended. |
| P14 | **T1:** Screenless attentional reset and environmental management. **T2:** Attention moved from model output to upstream input, followed by data cleaning and renewed analysis. | **T3:** Similar ambient music impeded complex English-language methods reading. **T4:** Whether the previous loop had ended; stop before precision work. **Timing:** Bounded; designed to be ignored. |
| P15 | **T1:** Embodied/affective attunement for expression. **T2:** Breathing and remembered bowing softened tension; earlier writing was retrieved and reorganised. | **T3:** The same embodied familiarity disrupted categorical art-history memory. **T4:** Hand-to-pen impulse versus deeper passive immersion; silence or performance alternatives. **Timing:** Bounded/phase-sensitive; active playing is a boundary case. |

### **B6. Analytic decision log**

| **Earlier candidate or analytic problem** | **Final decision** | **Rationale and evidence that prompted the decision** |
| --- | --- | --- |
| ‘Music as mood repair’ | Rejected as an overarching theme; replaced by the broader notion of **giving music a job**. | P02, P12 and P14 explicitly framed music as environmental or attentional management rather than emotional improvement. P05 sought activation, P09 sought pacing and P11 sought perceptual opening. ‘Mood repair’ would have erased these functions. |
| Separate candidates for ‘brief reset’ and ‘background enclosure’ | Brought together within Theme 1 but retained as contrasting forms of placement. | Both represented deliberate configuration of music at a friction point, yet their temporal architectures differed. P01/P03/P05/P06/P10/P13/P14 mainly bounded listening; P02/P04/P07/P09 often continued it. Keeping the distinction inside one theme enabled comparison without implying one correct method. |
| ‘Music-generated insight’ | Reworded as **altered access to a task representation** in Theme 2. | Participants repeatedly denied that music supplied answers: P01 called it a restart, P05 said it created room to re-examine the problem, P07 called it a buffer, P08 described clearing an assumption and P11 said an awareness was opened rather than told. The analytic claim was therefore changed from content production to enabling access. |
| Bodily response as a standalone therapeutic theme | Integrated into the transition sequence in Theme 2. | Bodily changes mattered because participants used them as signs that attention and action could change. Treating breathing or muscle release as an outcome in itself would overstate a therapeutic interpretation and disconnect it from learning re-engagement. |
| A feature list of ‘helpful music’ | Rejected in favour of **relational fit** in Theme 3. | Each apparent rule had a counterexample. Instrumental music could remain intrusive (P08/P12/P14); lyrics could be strategically activating or tolerable (P05/P07); familiarity could induce sleep or embodied capture (P05/P15); complexity could deliberately open creative possibility (P11). |
| A separate ‘harm’ theme for unhelpful listening | Integrated into Theme 3 as within-case reversals and into Theme 4 as monitoring information. | Negative episodes were analytically strongest when paired with the same participant’s helpful episode. They showed the boundary of fit and how later rules were learned, rather than forming one homogeneous category of harm. |
| A candidate map named after the stages of the emotion-regulation model | Not used as the final thematic structure. | The final order is compatible with identification, selection, implementation and monitoring, but themes were named through participants’ meanings. A theory-stage structure would have forced environmental masking, professional listening and active silence into categories that were too narrow. |
| ‘Finding the best music’ as the endpoint | Replaced with **regulating the regulator**, including silence. | All participants described stopping, switching or differentiating use. P08 and P13 interpreted headphone removal as task commitment, and P11 called active non-listening a musical decision. Adaptive withdrawal was therefore central rather than residual. |
| Musical expertise/active performance as a fifth theme | Retained as a cross-cutting boundary condition rather than a full-sample theme. | P06, P09, P11 and P15 showed that training increases both strategic sensitivity and distraction; P06, P09 and P15 also described humming or playing. These cases alter the interpretation of ‘music information’, but they do not warrant treating passive listening and active performance as equivalent across the sample. |

### **B7. Interpretive and reporting boundaries**

1. **The themes concern perceived process, not efficacy.** Terms such as described, experienced, attributed, associated with and made accessible are appropriate. The interview data do not demonstrate that music caused cognitive flexibility, reduced physiological stress or improved academic performance.

2. **Re-engagement was defined proximally.** A student had to report a task-relevant action—resuming, continuing, restructuring, searching, recalculating or changing strategy. Feeling calmer without action was not equated with learning engagement.

3. **Music and the micro-break were often inseparable.** Resting posture, breathing, water, movement, visual disengagement and rereading occurred alongside listening. The analysis retained this combination rather than assigning all change to the sound alone.

4. **Participant explanations remained attributed.** References to ‘working memory’, ‘language channels’ or related cognitive terminology were treated as students’ own sense-making. They were not reported as measured cognitive mechanisms.

5. **Coverage was descriptive, not quantitative.** The matrix was used to inspect whether a theme travelled across cases, how it changed form and where it failed. It was not used to estimate prevalence, impose a minimum participant count or rank themes by frequency.

6. **Negative cases were constitutive of the analysis.** Each participant’s unhelpful or counterproductive incident was used to refine the same thematic account rather than appended as an exception after a benefit narrative had been fixed.

7. **Silence and non-musical alternatives were coded as action.** Turning music off, using earplugs, moving, reading aloud or changing the work method were active regulatory choices, not missing data.

8. **The scope remained self-directed music use rather than clinical music therapy.** Some accounts approached acoustic masking or active music-making. These were retained as boundary cases and named transparently rather than folded into a uniform therapeutic intervention.

9. **Translation followed analysis.** The Mandarin source, English rendering and source locator for manuscript quotations are documented in Appendix C. Translation preserved conceptual and affective meaning but remains an additional interpretive layer.

**Appendix C. Bilingual Key-Quotation Audit Trail**

## **Purpose and handling rules**

This audit trail links the four Findings themes to 16 traceable quotations. Each Chinese quotation is a continuous, verbatim excerpt from the labelled transcript paragraph. English renderings use British English and preserve the analytic metaphor or contrast used in the Findings. Filler, named music, named software, course titles, locations and other potentially identifying detail are excluded by selecting a shorter continuous span; nothing inside a quoted span has been altered or silently deleted. No song lyric is reproduced. Participant codes (P01–P15) are the only identifiers.

"Helpful", "counterproductive", "mixed" and "boundary/contrast" describe the participant's account of the episode; they do not establish a causal effect.

## **Quotation index**

| **ID** | **Theme** | **Participant** | **Event type** |
| --- | --- | --- | --- |
| C01 | Theme 1 | P05 | Helpful—activation goal |
| C02 | Theme 1 | P02 | Boundary/contrast—soundscape management |
| C03 | Theme 1 | P13 | Helpful—reduce competing possibilities |
| C04 | Theme 1 | P12 | Helpful—interrupt indecision |
| C05 | Theme 2 | P06 | Helpful—body-to-cognition pathway |
| C06 | Theme 2 | P04 | Helpful—cognitive reframing |
| C07 | Theme 2 | P09 | Helpful—sustained task action |
| C08 | Theme 2 | P01 | Helpful—transition, not task substitution |
| C09 | Theme 3 | P01 | Counterproductive—salient new music |
| C10 | Theme 3 | P10 | Counterproductive—task-state mismatch |
| C11 | Theme 3 | P14 | Boundary/contrast—backgroundability |
| C12 | Theme 3 | P05 | Counterproductive—sleep loss |
| C13 | Theme 4 | P03 | Mixed/monitoring—regulation versus delay |
| C14 | Theme 4 | P13 | Helpful/monitoring—active closure |
| C15 | Theme 4 | P11 | Boundary—strategic silence |
| C16 | Theme 4 | P08 | Helpful/monitoring—stopping as start signal |

## **Theme 1. Giving Music a Job at Moments of Friction**

### **C01 — Activation rather than soothing**

• **Theme / subtheme / RQ:** Theme 1; Subtheme 1.2, music as an affective “tuner”; RQ1.

• **Participant code:** P05.

• **Event type:** Helpful episode; clearly specified activation goal.

• **Minimum anonymised context:** Before a demanding technical task, the participant sought alertness rather than calm.

• **Chinese verbatim:**

我不需要被安抚，我需要被激活。

• **Manuscript-aligned BrE translation:**

I do not need to be soothed; I need to be activated.

• **Translation / anonymisation note:** Direct semantic translation; the antithesis and passive construction are retained. This is a continuous sentence from the source. The surrounding genre, study location and equipment details are not quoted.

### **C02 — Environmental management rather than a universal emotion strategy**

• **Theme / subtheme / RQ:** Theme 1; Subtheme 1.2, music as an acoustic shield; RQ1, with a boundary relevant to RQ3.

• **Participant code:** P02.

• **Event type:** Boundary/contrast case; helpful soundscape management with an explicitly recognised failure condition.

• **Minimum anonymised context:** In a noisy shared study setting, the participant used sound to create an acoustic boundary but recognised that it could obstruct self-recitation.

• **Chinese verbatim:**

对我来说，它不像是一种“情绪调节”，更像是一种“环境管理”。我不是为了开心才听的，我是为了“屏蔽”。它是我把自己从嘈杂的物理世界里拽出来，放进一个只有我和题目的空间里的那扇门。但我也清楚，这扇门有时候会挡在错误的地方，比如挡住我自己念书的声音。

• **Manuscript-aligned BrE translation:**

For me, it was less a form of “emotion regulation” and more a form of “environmental management”. I did not listen in order to become happy; I listened in order to “block things out”. It was the door through which I pulled myself out of the noisy physical world and into a space containing only the problem and me. Yet I also knew that the door could block the wrong thing, such as my own voice when I needed to recite.

• **Translation / anonymisation note:** Continuous four-sentence excerpt; the “door” and “blocking” metaphors are retained. A discourse marker and the later general statement about the strategy not being universal fall outside the selected span; no content within the excerpt is omitted. No place, institution or third-party detail appears.

### **C03 — Clearing an overloaded mental workspace**

• **Theme / subtheme / RQ:** Theme 1; Subthemes 1.1 and 1.2, recognition of cognitive friction and assigning music an interruptive job; RQ1.

• **Participant code:** P13.

• **Event type:** Helpful episode; convergence goal.

• **Minimum anonymised context:** A long-form literature review had stalled among too many possible organising lines.

• **Chinese verbatim:**

我当时希望音乐能帮我“清理一下桌面”。不是指物理桌面，是我心里的桌面——那些太多太碎的想法需要被清掉一些，只留下最重要的那一条。

• **Manuscript-aligned BrE translation:**

I wanted music to help me “clear the desktop”. I did not mean the physical desktop, but the one in my mind—there were too many fragmented thoughts, and some needed to be cleared away so that only the most important thread remained.

• **Translation / anonymisation note:** Continuous two-sentence excerpt. “Desktop” and “the most important thread” retain the participant’s spatial and organisational metaphors rather than replacing them with psychological terminology. The specific degree programme and project topic are absent.

### **C04 — Stopping an internal argument**

• **Theme / subtheme / RQ:** Theme 1; Subtheme 1.2, music as a reset for an unproductive loop; RQ1.

• **Participant code:** P12.

• **Event type:** Helpful episode; interruption goal.

• **Minimum anonymised context:** The participant was oscillating between two possible routes through a technical problem.

• **Chinese verbatim:**

我当时其实希望音乐能帮我“停止内部争论”。因为我的问题不是算不出来，是“不知道选哪条路”。

• **Manuscript-aligned BrE translation:**

At that point, I wanted music to help me “stop the internal argument”. My problem was not that I could not calculate it; it was that I “did not know which route to choose”.

• **Translation / anonymisation note:** Continuous two-sentence excerpt. “Internal argument” and “route” are retained as participant metaphors. The discipline, course and formulae are excluded by stopping before the task-specific account.

## **Theme 2. Music Made the Transition; Students Did the Academic Work**

### **C05 — Bodily change opened a different way of thinking**

• **Theme / subtheme / RQ:** Theme 2; Subthemes 2.1 and 2.2, body-to-cognition pathway and cognitive reopening; RQ2.

• **Participant code:** P06.

• **Event type:** Helpful episode; participant-perceived indirect pathway.

• **Minimum anonymised context:** A written explanation had stalled while the participant remained physically tense.

• **Chinese verbatim:**

是那个呼吸和哼唱让我的身体状态先变了，然后我的思维方式才跟着变了。如果我一直在那“僵着”，我是想不到换个角度写的。所以音乐做的不是“给灵感”，而是“打开通道”。

• **Manuscript-aligned BrE translation:**

The breathing and humming changed my bodily state first, and then my way of thinking followed. If I had stayed tense like that, I would not have thought of writing from another angle. What music did was not to “give me inspiration”, but to “open a channel”.

• **Translation / anonymisation note:** Continuous three-sentence excerpt beginning after the participant’s brief qualification that the connection was indirect. “Tense” preserves the bodily sense of 僵着, while “open a channel” retains the source metaphor. No named text, task topic or music appears.

### **C06 — From negative self-judgement to problem identification**

• **Theme / subtheme / RQ:** Theme 2; Subtheme 2.2, attention moves from self-evaluation to a tractable problem; RQ2.

• **Participant code:** P04.

• **Event type:** Helpful episode; cognitive reframing.

• **Minimum anonymised context:** The participant was repeatedly judging and revising a report section.

• **Chinese verbatim:**

想法变化最明显——从“我写得好烂”变成了“这里只需要调结构就行”，视角从“自我否定”切换到“问题定位”。

• **Manuscript-aligned BrE translation:**

The clearest change was in my thinking—from “What I have written is terrible” to “I only need to adjust the structure here”. My perspective shifted from negative self-judgement to identifying the problem.

• **Translation / anonymisation note:** One continuous sentence. The colloquial intensity of 好烂 is rendered as “terrible”, and the analytic contrast is preserved. The report topic and location are absent.

### **C07 — External rhythm sustained academic action without supplying content**

• **Theme / subtheme / RQ:** Theme 2; Subtheme 2.3, sustained and observable task action; RQ2.

• **Participant code:** P09.

• **Event type:** Helpful episode; music continued during a compatible writing task.

• **Minimum anonymised context:** The participant was maintaining forward movement during a familiar writing task.

• **Chinese verbatim:**

音乐在那次的作用更像是“提供稳定的外部节律”。它没有给我任何内容上的贡献，但它让我在写的过程中持续保持一个稳定的输出节奏，不会因为卡壳就停下来很久。

• **Manuscript-aligned BrE translation:**

Music acted more like a “stable external rhythm”. It contributed nothing to the content, but it allowed me to maintain a steady pace of output while writing, rather than stopping for a long time whenever I became stuck.

• **Translation / anonymisation note:** Continuous two-sentence excerpt. “External rhythm” and “pace of output” retain the participant’s production metaphor. The later autobiographical account of instrumental practice is excluded, removing unnecessary personal detail.

### **C08 — A restart, not outsourced academic work**

• **Theme / subtheme / RQ:** Theme 2; Subtheme 2.3, observable re-engagement rather than general mood improvement; RQ2.

• **Participant code:** P01.

• **Event type:** Helpful episode; transition to resumed work.

• **Minimum anonymised context:** A technical task had stalled; after the listening interval, the participant resumed the task.

• **Chinese verbatim:**

音乐发挥的作用更像是一个“重启键”，它没有帮我写报告，但它帮我从那种卡死的状态里出来了。

• **Manuscript-aligned BrE translation:**

Music worked more like a “restart button”. It did not write the report for me, but it helped me out of that frozen state.

• **Translation / anonymisation note:** One continuous sentence. “Restart button” is retained; 卡死 is rendered as “frozen” to avoid introducing a technical diagnosis. The named software and specific error in the surrounding paragraph are excluded.

## **Theme 3. Fit, Not Genre, Determined Whether Music Helped**

### **C09 — Salience compounded anxiety with distraction**

• **Theme / subtheme / RQ:** Theme 3; Subtheme 3.2, perceptual salience and novelty; RQ3 and the hindering side of RQ2.

• **Participant code:** P01.

• **Event type:** Counterproductive episode.

• **Minimum anonymised context:** A new, attention-grabbing song was selected during a difficult study episode.

• **Chinese verbatim:**

它没有帮助我脱离压力，反而让我从“焦虑”变成“焦虑+分心”。

• **Manuscript-aligned BrE translation:**

It did not help me escape the pressure; instead, it turned “anxiety” into “anxiety plus distraction”.

• **Translation / anonymisation note:** One continuous sentence. The participant’s compact additive formulation is retained. The song, course and location are not named.

### **C10 — The same state helped association but hindered memorisation**

• **Theme / subtheme / RQ:** Theme 3; Subtheme 3.1, task processing demand; RQ3 and the hindering side of RQ2.

• **Participant code:** P10.

• **Event type:** Counterproductive episode paired with a previously helpful use of the same music.

• **Minimum anonymised context:** The same piece was used first for associative writing and later for precise memorisation.

• **Chinese verbatim:**

同一首音乐把脑子调成了适合联想的状态，但它正是背东西的时候最不需要的状态。

• **Manuscript-aligned BrE translation:**

The same piece of music put my mind into a state suited to making associations, but that was precisely the state I did not need when trying to memorise material.

• **Translation / anonymisation note:** One continuous sentence. The causal-sounding 调成 is rendered within the participant’s first-person account; the surrounding prose must continue to frame it as a perceived effect. The specific subject matter is omitted.

### **C11 — Useful background sound had to remain ignorable**

• **Theme / subtheme / RQ:** Theme 3; Subtheme 3.2, perceptual salience and backgroundability; RQ3.

• **Participant code:** P14.

• **Event type:** Boundary/contrast account; the feature defining potentially helpful sound also defines failure.

• **Minimum anonymised context:** The participant described the kind of low-salience audio that could be tolerated around selected study tasks.

• **Chinese verbatim:**

它不是用来“听”的，它的目的是“被忽略”。如果一首歌不能被忽略，它就失败了。

• **Manuscript-aligned BrE translation:**

It is not there to be “listened to”; its purpose is to “be ignored”. If a piece cannot be ignored, it has failed.

• **Translation / anonymisation note:** Continuous two-sentence excerpt. The passive contrast between attending and ignoring is retained. The surrounding discipline, task, location and audio examples are excluded.

### **C12 — Fatigue reversed familiar music into a sleep cue**

• **Theme / subtheme / RQ:** Theme 3; Subtheme 3.3, personal capacity and fatigue; RQ3.

• **Participant code:** P05.

• **Event type:** Counterproductive episode.

• **Minimum anonymised context:** Familiar music was used after insufficient sleep, when the participant’s alertness was already low.

• **Chinese verbatim:**

如果自己已经困了，音乐是救不了你的，它只会让你更舒服地睡着。

• **Manuscript-aligned BrE translation:**

Music cannot rescue you when you are already sleepy; it only lets you fall asleep more comfortably.

• **Translation / anonymisation note:** One continuous conditional sentence. The second-person form reflects a general lesson stated by the participant. The surrounding details about drinks and location are excluded.

## **Theme 4. Using Music Well Also Meant Knowing When to Stop**

### **C13 — A second song could signal avoidance**

• **Theme / subtheme / RQ:** Theme 4; Subthemes 4.1 and 4.3, real-time monitoring and the risk of delay; RQ3.

• **Participant code:** P03.

• **Event type:** Mixed/monitoring account; prospective counterproductive boundary.

• **Minimum anonymised context:** The participant used the desire for a second song as a self-check after a brief listening interval.

• **Chinese verbatim:**

听完一首歌（大概四到五分钟）之后，我如果还想继续听第二首，那就说明我还在“逃避”任务，这时候音乐是在帮我拖延；但如果我听了一首之后，我自己主动想停下来去翻书了，那就说明音乐已经帮我把门打开了。

• **Manuscript-aligned BrE translation:**

After one song—about four or five minutes—if I still want to listen to a second one, that means I am still “avoiding” the task, and the music is helping me procrastinate. But if, after one song, I want to stop of my own accord and open the book, that means the music has opened the door for me.

• **Translation / anonymisation note:** Continuous sentence from the middle of the source paragraph. Parentheses are rendered as an em-dash for idiomatic English; “avoiding”, “procrastinate” and the “door” metaphor preserve the source contrast. Later biographical and course-comparison details are excluded.

### **C14 — Removing the headphones as a commitment**

• **Theme / subtheme / RQ:** Theme 4; Subtheme 4.2, active stopping as part of the strategy; RQ3.

• **Participant code:** P13.

• **Event type:** Helpful/monitoring account; deliberate closure.

• **Minimum anonymised context:** The participant actively ended a pre-study listening ritual rather than allowing the playlist to end by itself.

• **Chinese verbatim:**

那个“摘耳机”的动作对我来说其实是一个心理上的commitment——我在告诉自己“我现在开始认真做事了”。

• **Manuscript-aligned BrE translation:**

For me, the act of “taking off my headphones” is a psychological commitment—I am telling myself, “I’m starting serious work now.”

• **Translation / anonymisation note:** One continuous sentence. The English word commitment already appears in the Mandarin source and is retained; the surrounding playlist length and room context are omitted.

### **C15 — Non-listening as an active musical decision**

• **Theme / subtheme / RQ:** Theme 4; Subtheme 4.2, strategic silence; RQ3.

• **Participant code:** P11.

• **Event type:** Boundary case; deliberate non-use following monitoring.

• **Minimum anonymised context:** The participant described silence as necessary when external sound would occupy internal auditory space.

• **Chinese verbatim:**

“主动选择不听”也是一个重要的音乐决策。

• **Manuscript-aligned BrE translation:**

“Actively choosing not to listen” is also an important musical decision.

• **Translation / anonymisation note:** One continuous sentence with the participant’s emphasis retained. The quotation contains no identifying detail; the broader specialist context is supplied only in anonymised form above.

### **C16 — The decision to stop marked task entry**

• **Theme / subtheme / RQ:** Theme 4; Subthemes 4.1 and 4.2, a behavioural monitoring cue and active stopping; RQ3, with an outcome link to RQ2.

• **Participant code:** P08.

• **Event type:** Helpful/monitoring account; active cessation.

• **Minimum anonymised context:** The participant reflected on deliberately ending a short listening interval before resuming a demanding task.

• **Chinese verbatim:**

不是“听了音乐才进入状态”，而是“决定关掉音乐”的那一刻，才是真正进入了状态。

• **Manuscript-aligned BrE translation:**

It is not listening to music that puts me into work mode; I only truly enter it at the moment I decide to turn the music off.

• **Translation / anonymisation note:** One continuous sentence. The repeated 状态 is rendered idiomatically as “work mode” and the pronoun “it”, without adding a new mechanism. No task-specific or identifying detail is included.

## **Cross-check against the Findings narrative**

| **Theme** | **Representative evidence** | **Contrast / negative evidence** | **Coverage supplied by this appendix** |
| --- | --- | --- | --- |
| Theme 1 | P05 activation; P13 convergence; P12 interruption | P02 rejects a universal emotion-regulation account and identifies a blocking risk | Four participants; three distinct assigned “jobs” plus a conceptual boundary case |
| Theme 2 | P06 body-to-cognition; P04 reframing; P09 sustained output; P01 restart | P01 and P09 explicitly deny that music supplied academic content | Four participants; bodily, attentional, cognitive and behavioural links represented |
| Theme 3 | P14 defines useful sound by its backgroundability | P01 salience, P10 task-state mismatch, P05 fatigue reversal | Four quotations covering musical salience, task demand and initial state |
| Theme 4 | P13 and P08 active stopping; P11 strategic silence | P03 identifies continued listening as potential avoidance | Four participants; monitoring, closure, silence and delay risk represented |

## **Audit limitations**

• The English translations are meaning-oriented and close to the Mandarin syntax; they are author translations checked for conceptual and affective equivalence as described in the Methodology, not independently back-translated versions.

• Minimal context has been deliberately generalised; the confidential raw transcript remains the authoritative source for the full episode.

• The selected quotations illustrate thematic range and negative cases; they are not frequency estimates or evidence of music's causal effects on cognition, learning outcomes or mental health.

• The P11 specialist excerpt was shortened in the Findings to prevent inferential identification; the body retains only the phrase “how much 'auditory blankness' the task required” and paraphrases the contrast at a broader level.
